# Survey of UK health professionals supporting parents after loss from a twin pregnancy

**DOI:** 10.1186/s12884-021-03543-9

**Published:** 2021-01-14

**Authors:** Judith Rankin, Louise Hayes, Nicholas Embleton

**Affiliations:** 1 Population Health Sciences Institute, Newcastle University, Baddiley-Clark Building, Richardson Road, Newcastle upon Tyne, NE2 4AX UK; 2 Newcastle upon Tyne Hospitals NHS Foundation Trust, Neonatal Unit, Richardson Road, Newcastle upon Tyne, UK

**Keywords:** Bereavement, Twin pregnancy, Health professionals, Survey

## Abstract

**Background:**

Bereavement from a twin pregnancy can result in complex emotions as parents are often caring for a surviving sibling while mourning the loss of their infant. Health professionals have reported feeling ill-equipped to deal with the specific needs of parents in this situation. Our aim was to ascertain the current knowledge, training needs and self-rated confidence of health professionals in providing support to parents who have experienced a loss from a twin pregnancy.

**Methods:**

We used an online survey, sent by email via professional organisations and clinical networks, to neonatal and fetal medicine doctors, neonatal nurses, and midwives. Respondents provided anonymous responses to questions on their experience of training and knowledge of existing guidelines, confidence in supporting parents and current practice in their hospital neonatal unit.

**Results:**

We received 293 responses. Less than half (47.3%) of respondents had received training for supporting parents and 62% felt more training and further guidelines were required. Less than a third of respondents reported having no or some confidence when providing emotional support to parents.

**Conclusions:**

Current training and guidelines in the UK to support health professionals caring for parents who have experienced a loss from a twin pregnancy are inadequate. Guidelines for healthcare professionals who support parents experiencing the loss of a baby from a twin pregnancy are needed.

**Supplementary Information:**

The online version contains supplementary material available at 10.1186/s12884-021-03543-9.

## Background

The loss of a baby is a traumatic and significant event that remains with the parents for a lifetime. The importance of sensitive emotional care from health professionals at the time of the loss of their baby is well documented as having a significant impact upon life-long memories of the experience formed by parents, grief and psychological distress [1]. What is less well known is how confident health professionals feel in providing this important emotional care and whether sufficient training is available to support them to do so.

Bereavement from a twin pregnancy can result in particularly complex emotions as parents are often caring for a surviving sibling who may not be well while mourning the loss of their infant [2]. In our previous research on loss from a twin pregnancy, mothers highlighted the importance of the emotional support they received from health professionals whilst their surviving baby was being cared for; participants stressed how highly they valued health professionals who acknowledged and showed sensitivity to their loss [2]. On the other hand, health professionals reported feeling ill-equipped in terms of time, resources and expertise to deal with the specific needs of parents in this situation and specifically called for more training [3].

The aim of this study was to ascertain the current knowledge, training needs and self-rated confidence in providing care to parents who have experienced a loss from a twin pregnancy, of health professionals working in obstetrics, fetal medicine and neonatology.

## Methods

We developed a web-based survey (see Supplementary material) for health professionals involved in supporting parents who have experienced a loss from a twin pregnancy whilst caring for a surviving sibling. The survey included fixed and open ended questions on current knowledge, practice and self-rated confidence when caring for parents who have experienced a loss. For the fixed questions, respondents selected from responses provided. For ‘confidence’ these were on a 4 point scale from ‘No confidence’ to ‘Very confident’ and these were subsequently grouped into ‘No or some confidence’ and ‘Fairly or very confident’. For ‘view of how satisfactorily parents are supported’, these were on a 4 point scale from ‘Not very’ to ‘Very well’. Respondents were also asked to indicate their ‘confidence in supporting parents compared to their confidence in other roles they undertake’. Responses to these questions were on a 3 point scale from ‘Less confident’ to ‘More confident’. We also asked about their training needs; in this context training referred to any professional development they thought they would benefit from. The questions were developed by the authors and were informed by our previous research [3]. The survey was piloted with a representative from each of the relevant specialties involved. The survey was distributed nationally within the UK by email via professional bodies (the British Association of Perinatal Medicine, the British Maternal and Fetal Medicine Society, the Neonatal Network and the Royal College of Paediatrics and Child Health), and leading charities in the field (the Multiple Birth Foundation and Bliss). The survey ran for 4 months in 2016; one reminder was sent. All responses were anonymous.

The number (and percentage) of respondents is given. Descriptive statistics are presented. Chi-square tests were used to test for differences in knowledge, training and confidence between respondents by experience in their role and between different professional groups (doctor, nurse, midwife). Analyses were conducted using SPSS V22.0 [4]. As there were a number of quotes for each open ended question, we have had to be selective in choosing which quotes to present here. We read through all quotes for each question and include those that best illustrate the theme under discussion.

## Results

We received a total of 293 responses from those working in the UK. We are not able to provide a response rate as the survey was disseminated on our behalf and the organisations did not collect this information. Of these respondents, 232 (79.5%) were female, 168 (57.5%) were over the age of 45 years and 214 (83%) had been working in their current role for over 10 years (Table 1). Just over half of respondents worked in neonatal medicine (156, 53.2%) and 199 respondents (67.9%) worked in a hospital with a neonatal intensive care unit (NICU).

**Table 1:** Summary characteristics of survey respondents

	N (total *n* = 293)	%
**Gender**		
Male	60	20.5
Female	232	79.5
*(1 missing)*		
**Age group**		
< 45 years	124	42.5
> 45 years	168	57.5
*(1 missing)*		
**Years in role**		
< 5 years	22	8.5
5–10 years	22	8.5
10–15 years	43	16.7
15+ years	171	66.3
*(35 missing)*		
**Role**		
Doctor	137	51.7
Nurse	80	30.2
Midwife	48	18.1
*(28 missing)*		
**Specialty**		
Neonatal medicine	149	50.9
Fetal medicine	127	43.3
Other	17	5.8
**Main place of work**		
Hospital with a neonatal intensive care unit	199	67.9
Hospital with a small baby unit	80	27.3
Community or primary care	5	1.7
Other	9	3.1

### Training and knowledge of guidelines on loss from a twin pregnancy

Just under half of the respondents (131, 47.3%) reported receiving training on loss from a twin pregnancy (Table 2). Ninety-one (32.9%) respondents had used national guidelines to inform their practice and 103 (37.2%) knew of local guidelines. A third of respondents (91, 33%) felt that the current training offered and current guidelines available were inadequate. A further 172 (62.3%) respondents felt that more training and guidelines would be helpful. This is further evidenced through the following illustrative quotes:

‘*Would be so grateful for any information as I lead the bereavement team and this has made me really think that we need to offer more support and teaching to our staff in this area.’* (Neonatal nurse).

*‘I feel like there are probably guidelines out there but I think staff need to be more aware.’* (Neonatal nurse).

**Table 2:** Training around, and knowledge of, guidelines related to loss from a twin pregnancy

		N	%
**Received training** *(16 missing)*	**Yes**	131	47.3
**Used national guidelines** *(16 missing)*	**Yes**	91	32.9
**Know of local guidelines** *(16 missing)*	**Yes**	103	37.2
**Opinion of current training/guidelines**			
Inadequate		91	33.0
More training/guidelines would be helpful		172	62.3
Adequate		13	4.7
*(17 missing)*			
**Number of times supported parents in past year**			
Never		12	4.5
Not directly		49	16.7
1–2 sets of parents		110	37.5
More than 2 sets of parents		94	32.1
*(28 missing)*			
**Providing practical support**			
No or some confidence		109	37.2
Fairly or very confident		156	53.2
*(28 missing)*			
**Providing emotional support**			
No or some confidence		80	27.3
Fairly or very confident		185	69.8
*(28 missing)*			
**Confidence compared to providing medical care**			
Less confident		77	30.1
Equally confident		136	53.1
More confident		43	16.8
*(37 missing)*			
**Confidence compared to singleton loss**			
Less confident		60	22.8
Equally confident		199	75.7
More confident		4	1.5
*(30 missing)*			
**How well does continuity of care occur**			
Not very well/satisfactory		96	36.5
Fairly/very well		167	63.5
*(30 missing)*			
**How well are parents prepared for discharge**			
Not very well/satisfactory		81	31.0
Fairly/very well		180	69.0
*(32 missing)*			

### Providing practical and emotional support to parents

A total of 204 (71%) respondents had supported parents who had experienced the loss of a baby from a twin pregnancy on at least one occasion in the previous year. Just over half of respondents (156, 58.9%) were fairly or very confident about providing practical support when caring for parents who had experienced a loss from a twin pregnancy. However, almost a third of respondents reported feeling less than ‘fairly confident’ when providing emotional support to parents in this situation. Compared to providing medical care, 77 (30.1%) respondents were less confident providing emotional support, 136 (53.1%) were equally as confident and 43 (16.8%) respondents reported feeling more confident. Sixty (22.8%) respondents were less confident in providing emotional support when the loss was from a twin pregnancy than if the loss was a singleton, 199 (75.7%) reported feeling equally as confident and four (1.5%, all were neonatal staff) said they felt more confident. Just over a third of respondents (96, 36.5%) said that continuity of care was not good or satisfactory and 167 (63.5%) respondents reported that continuity of care is done fairly or very well.

*‘I think we quickly forget that the baby we care for is a surviving twin, as soon as the baby has a first name we omit to communicate in any way that this is a twin multiple pregnancy and parents are treated in the same way as those who had a singleton pregnancy. We have guidelines when there is a neonatal death but there are not specific to the loss of a twin’* (Neonatal nurse)**.**

‘*Hugely under-appreciated area of practice. The emotional burden on couples is immense.’* (Fetal medicine consultant).

‘*We have some bereavement education sessions with staff in our unit and we thought we were okay with providing these services until the time when we had two couples each losing two and one of the triplets. And then we were lost...could not apply the described rules. the biggest difference in facing parents of singleton losses versus multiple fetuses losses is - facing them daily when they come regularly to the unit to visit their surviving baby/ies- how much is more and how little is too little- confusion prevails!!’* (Specialist registrar, neonatology).

This quote highlights the discrete nature of interactions of health professionals with parents who have experienced a singleton loss that is different when the loss has been from a multiple pregnancy. Health professionals referred to how difficult they found this emotional work in our previous qualitative work [3].

### Training and confidence by experience

Table 3 shows the results from the questions on training and confidence in providing practical and emotional care by experience (number of years in role). Those with > 10 years in their role were more likely to have received training compared to those with < 10 years (X^2^ = 11.189; df = 3; *p* = 0.011). Those respondents with < 10 years’ experience reported no or some confidence in providing practical support to parents who had experienced a loss from a twin pregnancy (X^2^ = 31.326; df = 3; *p* = 0.000). In terms of emotional support, more respondents with less experience reported no or some confidence in providing emotional support compared to those with more years’ experience ((X^2^ = 20.194; df = 3; *p* = 0.000). There were no significant differences by experience in any of the other training and confidence questions.

**Table 3:** Training around, and knowledge of, guidelines related to number of years worked in setting where loss occurs

	Years in role				
	< 5 years N (%)	< 10 years N (%)	10–15 years N (%)	> 15 years N (%)	
**Received training**	6 (28.6)	5 (23.8)	21 (51.2)	89 (54.9)	X^2^ = 11.189; *p* = 0.011
**Used national guidelines**	9 (42.9)	4 (19.0)	16 (39.0)	52 (32.1)	X^2^ = 3.501; *p* = 0.321
**Know of local guidelines**	7 (33.3)	6 (28.6)	14 (34.1)	64 (39.5)	X^2^ = 1.37; *p* = 0.720
**Opinion of training/guidelines**					
Inadequate	9 (42.9)	9 (42.9)	18 (43.9)	39 (24.1)	X^2^ = 10.533 *p* = 0.014
More would be helpful	11 (52.4)	12 (57.1)	21 (51.2)	113 (69.8)	
Adequate	1 (4.8)	0	2 (4.9)	10 (6.2)	
**Providing practical support**					
No or some confidence	13 (76.5)	16 (76.2)	20 (50.0)	45 (28.5)	X^2^ = 31.336 *p* = 0.000
Fairly or very confident	4 (23.5)	5 (23.8)	20 (50.0)	113 (71.5)	
**Providing emotional support**					
No or some confidence	10 (58.8)	10 (47.6)	16 (40.0)	31 (19.6)	X^2^ = 20.194 *p* = 0.000
Fairly or very confident	7 (41.2)	11 (52.4)	24 (60.0)	127 (80.4)	
**Confidence providing medical care compared to emotional care**					
Less confident	6 (35.5)	10 (47.6)	14 (35.9)	35 (23.3)	X^2^ = 9.315 *p* = 0.155
Equally confident	10 (58.8)	7 (33.3)	18 (46.2)	89 (59.3)	
More confident	1 (5.9)	4 (19.0)	7 (17.9)	26 (17.3)	
**Compared to singleton loss**					
Less confident	6 (35.3)	6 (28.6)	13 (32.5)	31 (19.7)	X^2^ = 9.172 *p* = 0.164
Equally confident	10 (58.8)	15 (71.4)	26 (65.0)	125 (79.6)	
More confident	1 (5.9)	0	1 (2.5)	1 (0.6)	

### Training and confidence by role

Table 4 shows the results from the questions on training and confidence in providing practical and emotional care by clinical role. There was a significant difference between doctors and nurses in who had received training; fewer midwives had received training compared to doctors and nurses (X^2^ = 8.999; df = 2; *p* = 0.011). There was also a significant difference by clinical role in response to the question on confidence in providing emotional and practical care; more nurses than doctors and midwives said they were less or equally as confident to provide emotional care as practical care and more midwives reported being more confident (X^2^ = 40.143; df = 4; *p* < 0.001; Table 4).

**Table 4:** Training around, and knowledge of, guidelines related to role

		Doctor (%) (***n*** = 137)	Midwife (%) (***n*** = 48)	Nurse (%) (***n*** = 80)	p for difference
		N (%)	N (%)	N (%)	
**Received training**	Yes	70 (55.1)	14 (29.8)	35 (45.5)	X^2^ = 8.999; *p* = 0.011
**Used national guidelines**	Yes	40 (31.5)	13 (27.7)	25 (32.5)	X^2^ = 0.336; *p* = 0.845
**Know of local guidelines**	Yes	42 (33.1)	22 (46.8)	25 (32.5)	X^2^ = 3.263; *p* = 0.196
**Opinion of current training/guidelines**					
Inadequate		36 (28.6)	19 (40.4)	37.7	X^2^ = 8.259; *p* = 0.083
More would be helpful		79 (62.7)	27 (57.4)	47 (61.0)	
Adequate		11 (8.7)	1 (2.1)	1 (1.3)	
**Providing practical support**					
No or some confidence		42 (34.7)	18 (40.9)	39 (48.7)	X^2^ = 3.800 *p* = 0.150
Fairly or very confident		79 (65.3)	26 (59.1)	39 (51.3)	
**Providing emotional support**					
No or some confidence		40 (33.1)	11 (25.0)	24 (31.6)	X^2^ = 0.988; *p* = 0.610
Fairly or very confident		81 (66.9)	33 (75.0)	52 (68.4)	
**Compared to providing medical care**					
Less confident		35 (29.9)	7 (17.1)	28 (37.3)	X^2^ = 40.143; *p* < 0.001
Equally confident		66 (56.4)	14 (34.1)	44 (58.7)	
More confident		16 (13.7)	20 (48.8)	3 (4.0)	
**Compared to singleton loss**					
Less confident		29 (24.0)	13 (30.2)	12 (16.0)	X^2^ = 5.864; *p* = 0.210
Equally confident		92 (76.0)	29 (67.4)	62 (82.7)	
More confident		0	1 (2.3)	1 (1.3)	

## Discussion

This study describes current levels of training and confidence as reported by health professionals in providing practical and emotional care to parents who have lost a baby from a twin pregnancy. Less than half of respondents had received training on this important aspect of clinical care and the majority of respondents felt more training and further guidelines were needed. Whilst there was a high level of confidence reported in providing practical support when caring for parents in this situation, almost a third of respondents reported having little confidence when providing emotional support to parents. Continuity of care was also reported to be less than satisfactory by a third of respondents. For less experienced health professionals, confidence in providing emotional support was reported to be lower than for those with more experience.

We chose to use an online survey to capture responses as this provided a pragmatic approach to gather important information from a range of health professionals working in relevant specialties and in different environments. We acknowledge that there may have been other organisations that could have been relevant to include and that our approach only provided a snapshot of opinion and experience. There may be some bias in the responders, as to respond required access to the internet and also membership of the professional body or professional email distribution lists that we contacted. Although we provided the opportunity for respondents to give free text responses, there may be other relevant areas that we did not include in the survey. It has been shown that self-assessment has limitations with clinicians tending to underreport their skill level [5]. We divided age into two groups: over and under 45 years of age. This was a pragmatic decision based on the lower number of respondents in the younger age groups. We received more responses from females reflecting the overall gender distribution within these professions. However, we received responses from all of the relevant specialties (obstetrics, midwifery, fetal medicine and neonatology), from across the UK, from those working in different hospital settings and self-rated confidence in clinical and practical care provided to parents was high.

The available literature on bereavement care following loss from a twin pregnancy is scarce. However, existing studies recognise that parents who lose one twin have a complex set of emotional needs that may differ from those parents who lose a singleton [2]. The following review of studies, although relevant in relation to the training needs of health professionals working with bereaved parents, comes mainly from experiences of the loss of a singleton baby. An interview-based study with eight nurses in Canada found that communication with bereaved families can be particularly challenging and intimidating to nurses, especially for those with little experience [6]. A qualitative study of 19 health professionals (nurses, midwives, nursing auxiliaries and obstetricians) from one hospital in Córdoba, Spain, showed that health professionals may experience feelings of sorrow, anxiety, guilt, failure and helplessness related to not knowing how to deal with perinatal loss [7]. It was also common for professionals dealing with perinatal death to focus on physical care, thus avoiding the emotional aspects, in an attempt to decrease anguish [7]. In the present study, 26.3% of respondents felt less confident about providing bereavement support compared to providing medical care (14.7% felt more confident). It has been reported that in order to support families through bereavement, nurses need to confront the ‘negative social norms on death’. This includes the way in which they are supported by or confined by institutional practices [6]. Having an institutional policy for bereavement care is considered beneficial, as it ‘empowers’ the staff member [6]. Nurses who were surveyed in five hospitals in Hong Kong, perceived perinatal bereavement training to be important/very important. Nearly 90% of the nurses felt that training would equip them with relevant knowledge and skills to be able to support parents [8]. A further study in Hong Kong among 101 individuals who offered perinatal bereavement support found that having adequate training could help to increase levels of perceived self-confidence [9]. Past experience and age of the health professional is positively associated with a higher perceived self-confidence in caring for grieving parents [8, 9].

In the present study, approximately a third of the respondents felt that their current training/guidelines were inadequate and just over half reported that more training/guidelines would be helpful. In previous work, health professionals expressed a desire for more specialist training in supporting parents who had lost a baby from a twin pregnancy [3]. Following on from this work, and work with parents who have experienced twin pregnancy loss [2], we have developed guidelines for supporting health professionals dealing with bereavement from a twin pregnancy [10]. Positive behaviours and actions that staff can adopt reported to be appreciated by parents, as well as things that parents may find upsetting and insensitive, are summarised in the guidance. Although the guidelines focus on supporting parents, the importance of staff wellbeing in dealing with these challenging circumstances is also emphasised. It is recommended that provision for staff to reflect on the emotional impact of their work should be made within the NICU. This survey identified that health professionals with less years’ experience were likely to be less confident in and less likely to have had training for providing emotional support for parents. In 2018, The National Bereavement Care Pathway for Pregnancy and Baby Loss [11] was published; contained within it is advice for staff dealing with all types of loss. The following have been highlighted as important: Recognise their own support needs; Identify their own training needs; Communicate these needs to management and colleagues; Ensure they are aware of the support structures and systems in place within their Trust; Be aware of the stresses and challenges faced by colleagues and, where appropriate, flag support systems to them [11].

Staff awareness of their own needs and available support structures will undoubtedly contribute to confidence and coping skills in aiding parents experiencing loss. However, as raised in this study and in previous literature, there is a need for specific guidance in dealing with twin pregnancy loss. The guidelines developed as a result of this survey study and previous work [2, 3] aim to address this need [10, 12]. However, further research is needed to enable health professionals to understand more fully the complex needs of parents who experience the death of a twin in utero, at birth or in the neonatal period [3].

## Conclusion

Our survey showed that current training and guidelines in the UK to support health professionals caring for parents who have experienced a loss from a twin pregnancy are inadequate. Guidelines for healthcare professionals who support parents experiencing the loss of a baby from a twin pregnancy are needed.

## Supplementary Information

**Additional file 1.**

## Data Availability

All data generated or analysed during this study are included in this published article.

## Abbreviations

NICU: Neonatal Intensive Care Unit
UK: United Kingdom
