# Supplementary material for: Survey of UK health professionals supporting parents after loss from a twin pregnancy
Source: BMC Pregnancy Childbirth. 2021 Jan 14;21:58. doi: 10.1186/s12884-021-03543-9 (PMC7809801; doi:10.1186/s12884-021-03543-9)
Supplement: Supplementary file 1 — Additional file 1. [file 12884_2021_3543_MOESM1_ESM.pdf]

Loss from a multiple pregnancy - health professionals

Section one: About you

Families who have lost one or more babies from a multiple pregnancy face the difficult challenge of dealing with the bereavement, while often simultaneously feeling anxious about the prognosis for surviving babies. The practical and emotional support that staff provide to these parents can have a significant impact on their experience of this very difficult time. This situation may be encountered in antenatal clinics, on postnatal wards or on special care baby units (SCBUs), and in primary care settings e.g. community midwives, GPs or Health visitors. We are undertaking research in this area and would be very grateful if you could tell us how well equipped you feel you are to provide support to parents in this situation.

All the information that you provide in this questionnaire is given anonymously and will be treated as confidential data. We will write up the results in a scientific paper and they will be presented at a conference.

1. Are you male or female?

- ◯ Male
- ◯ Female
- ◯ Prefer not to say

2. How old are you?

- ◯ 18-24 years
- ◯ 25-34 years
- ◯ 35-44 years
- ◯ 45-54 years
- ◯ 55+

3. What is your job/role?

- ◯ Neonatal nurse
- ◯ Neonatal consultant
- ◯ Neonatal/paediatric ST
- ◯ Other neonatal staff
- ◯ Midwife
- ◯ Obstetrician
- ◯ Fetal medicine nurse
- ◯ Fetal medicine consultant
- ◯ Fetal medicine ST
- ◯ Nursing student
- ◯ Medical student

Other (please decribe)

4. How many years have you worked in a setting where this situation (loss of a baby from a multiple pregnancy) might occur?

In your current job?

In total?

5. What year did you complete your training?

6. What is your main place of work?

- ◯ Hospital with NICU (Level 3)
- ◯ Hospital with smaller baby unit
- ◯ Community or primary care

Other (please describe)

7. Which region do you work in (UK & ROI only)?

8. Place of work (if outside UK & ROI)

9. How did you hear about this survey?

- Internet search
- BAPM or RCPCH
- MBF
- Bliss
- Friend/colleague

Other (please give details)

Section two: Teaching, training and use of guidelines

The questions in this section refer to your experience of training and knowledge of guidelines related to supporting parents who have suffered a loss from a multiple pregnancy.

10. Have you been involved in any teaching/training sessions where this scenario has been discussed?

- ○ Yes
- ○ No

11. Have you used or accessed any national guidelines or resources related to this situation?

- ○ Yes
- ○ No

12. Do you have access to or know if local or departmental guidelines exist where you currently work?

- ○ Yes
- ○ No

13. In your current area of work, do you think existing teaching/training or guidelines meet the needs of staff and equip them to support parents who have suffered a loss from a multiple pregnancy?

- ○ No, current training and guidelines are inadequate
- ○ To some extent, but more training/guidelines would help
- ○ Current training and guidelines are adequate

14. Please give any other information about existing guidelines that you think might be of interest:

15. Are you aware of any existing guidelines around providing support for parents who have suffered a loss from a multiple pregnancy?

- ◯ Yes
- ◯ No

If yes, please give details (name of guideline and when and by whom produced, if known):

Loss from a multiple pregnancy - health professionals

Section three: Current practice around supporting parents

The following questions ask you to tell us how confident you feel in supporting parents after the death of one or more babies from a multiple pregnancy and about current practice in your unit. We are interested in losses that occur at any stage during the pregnancy or around the time of birth.

16. How many times in the past year have you cared for parents who have lost a baby from a multiple pregnancy?

- ◯ Never
- ◯ We have had parents like this, but I wasn't directly involved
- ◯ I have been involved with 1-2 sets of parents
- ◯ I have been involved with more than 2 sets of parents

17. How confident do you feel in providing practical support and information (for example, knowing how and when to refer parents for bereavement counselling, providing advice on birth and death registration, planning the funeral etc.)?

- ◯ No confidence
- ◯ Some confidence
- ◯ Fairly confident
- ◯ Very confident

18. How confident do you feel in providing emotional support and acknowledging the loss of a twin (for example, to what extent do you feel you have the confidence to ask parents about how they are feeling emotionally; do you have confidence in discussing the twin who died with the parents, mentioning them by name etc.; do you have confidence to support parents to make mementoes of their babies)?

- ◯ No confidence
- ◯ Some confidence
- ◯ Fairly confident
- ◯ Very confident

19. How well do you think continuity of care occurs in usual practice (for example ensuring communication between staff at handovers and between wards and units is good and ensuring parents are fully informed of any changes to treatment plans etc.)?

- Not very
- Satisfactorily
- Fairly well
- Very well

20. How well do you think parents are prepared for the discharge of their surviving twin from your unit?

- Not very well
- Satisfactorily
- Fairly well
- Very well

21. Please indicate how confident you feel in supporting parents who have suffered the loss of a baby from a multiple pregnancy in comparison to providing medical care for sick babies:

- Less confident
- Equally confident
- More confident

22. Please indicate how confident you feel in supporting parents who have suffered the loss of a baby from a multiple pregnancy in comparison to supporting parents who have suffered the loss of a singleton:

- Less confident
- Equally confident
- More confident

Loss from a multiple pregnancy - health professionals

Many thanks for taking the time to complete this survey. We really appreciate your input.

23. If you want to receive a pack or updates, and be informed of future developments of the project please give your email address below.

24. Name of your hospital or place of work, if you want to receive a pack or updates (so we don't send duplicate packs to the same unit)

25. Please provide any other comments on your experiences, knowledge or insights into this area
